# National Medical Journal

**Published:** 1872-03

**Authors:** 


### NATIONAL MEDICAL JOURNAL.

This journal has suspended, in consequence of a conflict of opinion between its editors and publishers. The editors—Drs. Busey and Lee—retired because the publishers took the responsibility, against their protest, of inserting a communication in the Journal.

In the circular of the publishers, "bad faith" is charged against the "late editors." We regret they should have so widely circulated a charge so damaging to the "late editors," should it be true, without the shadow of proof in its support. The profession should demand, for its own protection, and the character of the late editors, the establishment of the truth of the charge, or refuse, to a man, to have any editorial connection with that journal in the future. It would be a violation of the *spirit* of the code, should any professional man consent to assume the editorial management of that Journal, and thus endorse the charge, which may be false, and work serious injury to the character of his professional brethren.

---

### INFORMATION WANTED.

Dr. Robt. P. Harris, Pres. Phil. Obstet. Society, wishes to get a record of all cases of Cæsaro-hysterotomy which have occurred the United States. He has, as yet, only received one from Georgia, and desires all others to be reported to him, that Georgia surgeons may be duly credited in the tables he is preparing.

